# Evaluation of treatments for sacroiliitis in spondyloarthropathy using the Spondyloarthritis Research Consortium Canada scoring system

**DOI:** 10.1186/s13075-016-0916-2

**Published:** 2016-02-01

**Authors:** Yang Cui, Jinping Zheng, Xiao Zhang, Hui Zeng, Riqiang Luo

**Affiliations:** Department of Rheumatism, Guangdong General Hospital, No. 106, Zhongshaner Road, Guangzhou, China; Medical Imaging Centre, Guangdong General Hospital, Guangzhou, 510010 China

**Keywords:** spondyloarthritis, sacroiliitis, imaging studies, scoring, biologics, disease-modifying anti-rheumatic drugs

## Abstract

**Background:**

In this study, the Spondyloarthritis Research Consortium Canada (SPARCC) scoring method was used to compare treatment methods in patients with axial spondyloarthritis (SpA), a form of sacroiliitis. MRI abnormalities in bone marrow edema (BME) were compared before and after treatment in order to compare the efficacy of anti-TNF-α and DMARD, alone or in combination, as treatments for sacroiliitis.

**Methods:**

Fifty-six Chinese patients with axial SpA (mean age 22.6 years) were recruited. Patients were divided into three groups according to different treatments (anti-TNF-α alone vs. DMARDs alone vs. combined anti-TNF-α and DMARDs). MRI examinations were performed before and after treatment. The SPARCC score, clinically relevant AS Disease Activity (ASDAS) indices, erythrocyte sedimentation rate (ESR), and C-reactive protein (CRP) were analyzed.

**Results:**

After treatment, ASDAS and SPARCC scores, ESR, and CRP were significantly improved (P < 0.05) in the anti-TNF-α monotherapy and combination groups; however, there were no statistically significant differences (P > 0.05) in clinical disease activity and radiological inflammation of sacroiliac joint (SIJ) in patients in the DMARDs alone group. SPARCC showed a correlation with ASDAS score pre-treatment, but not post-treatment. Furthermore, there were significant changes (P < 0.05) in these patients with axial SpA after only 3 months of treatment. Follow-up studies of patients who continued therapy for 4-6 months and 9-12 months revealed statistically significant differences from baseline (P < 0.05).

**Conclusions:**

SPARCC can be used to assess severity of disease pre-treatment. Anti-TNF-α treatment resulted in effective reduction of disease activity and BME of SIJ after 3 months of therapy.

## Background

Spondyloarthritis (SpA) is a chronic, debilitating inflammatory rheumatic disease that affects axial and peripheral joints, organs, and other tissues. Epidemiology surveys of patients with SpA in China have reported a prevalence of SpA in the military of 0.45 %; however, the pooled incidence and prevalence of SpA from studies performed in civilian populations is 0.93 %. These numbers are similar to the prevalence found in Caucasian populations in Europe or the United States [1–11]. Sacroiliitis is the pathological sign and one of the early manifestations of SpA [12]. The management of SpA is extremely difficult. The traditional anti-rheumatic drugs (disease-modifying anti-rheumatic drugs (DMARDs)) are not clearly effective towards improving arthropathy of the central axis. In addition, numerous adverse reactions limit their clinical application. Biological agents such as tumor necrosis factor alpha (TNFα) inhibitor have been used in clinical research to treat ankylosing spondylitis (AS), showing efficacy against disease reactiveness and positive effects on joint function.

During the 2013 European League against Rheumatism (EULAR) meeting on spondyloarthropathy, it was determined that inflammation demonstrated by magnetic resonance imaging (MRI) and radiographic progression should be considered when determining therapeutic goals. Therefore, a standard assessment system is necessary for evaluating imaging changes in the sacroiliac joint (SIJ) in patients with SpA. In a study by Landewe et al. [13], the Spondyloarthritis Research Consortium Canada (SPARCC) system had a high level of inter-reader variability and sensitivity to change. The AS working group of the International Association for the Evaluation of Spinal Arthritis/Outcome Measures in Rheumatology (ASAS/OMERACT) proposed that MRI should be used as the first choice for evaluation of spinal arthritis [14].

More and more studies have used the SPARCC scoring system [15] to evaluate the efficacy of treatments in reducing joint inflammation of patients with SpA. Previous studies have shown a correlation between SPARCC scores and clinical disease activity. Zhang et al. [16] determined a correlation between the apparent diffusion coefficient (ADC) and SPARCC scores. However, little attention has been focused on the period in which bone marrow edema (BME) of the SIJ should be evaluated and the differences in joint inflammation changes between different therapies. Therefore, this study aimed to investigate the value of the SPARCC scoring system in evaluating the treatment of patients with axial SpA. Specifically, the efficacy of anti-TNFα and DMARDs as treatments for sacroiliitis was assessed with both drugs alone or in combination.

## Methods

### Patient eligibility

Fifty-six patients with SpA treated at the Guangdong General Hospital between September 2012 and March 2014 were recruited. Inclusion criteria included meeting the ASAS classification criteria for axial SpA [17]. The Bath Ankylosing Spondylitis Disease Activity Index (BASDAI) score had to be ≥4 [18]. No patient had been treated with systemic glucocorticoids for a period of 3 months prior to the trial. Participation required that patients had no active infectious diseases or severe organ function failure.

The 56 patients were divided into three groups according to therapy type. There were 18 patients with SpA treated using anti-TNFα alone, 21 patients treated using anti-TNFα combined with DMARDs, and 17 patients treated with DMARDs alone. All patients were allowed to use nonsteroidal anti-inflammatory drugs (NSAIDs) during the study as needed. The dose of anti-TNFα was gradually reduced as the disease became under better control (Ankylosing Spondylitis Disease Activity (ASDAS) indices <1.3) [19]. The reduction in dosage was as follows: if the patient’s ASDAS was <1.3 after 3 months of adequate therapeutic dosage of anti-TNFα, the interval between injections was extended (from 50 mg every week down to 50 mg every 10 days); and when ASDAS remained <1.3 after another 3 months of treatment, the interval was extended to 50 mg every 15 days. No patient discontinued treatment before the end of the study, and the dose for those who had undergone DMARD treatment remained the same as at the beginning of the study. Patients underwent MRI before and within 12 months after treatment.

Patients ever treated with anti-TNFα (total 39 patients, including anti-TNFα monotherapy and anti-TNFα combined with DMARDs) were divided into three subgroups according to the period of MRI examination: Group A, patients underwent MRI in their 3rd month of treatment (*n* = 10); group B, patients underwent MRI during their 4th–6th month of treatment (*n* = 17); and group C, patients underwent MRI during their 9th–12th month of treatment (*n* = 12).

The study was approved by the ethical committee of Guangdong General Hospital, China (approval No. 2013005H(RE)). Written informed consents were obtained from the participants.

### MRI scan

All patients’ SIJs were examined using T1-weighted images (T1WI), T2 fast-suppressed (T2-fs), and short-tau inversion recovery (STIR). The BME of the SIJ was scored by two experienced radiologists (10 and 2 years of experience, respectively) according to the SPARCC sacroiliitis scoring system. Readers were blinded to treatment and time sequence of the images. Final scores were taken as the average of the two readers.

### SPARCC scoring system

The radiologists observed an increased signal in the iliac bone and sacrum in six consecutive slices through the SIJ on T2-fs images or STIR sequences on oblique coronal slices. These slices comprise most of the synovial compartment. Each SIJ is divided into four quadrants, and each quadrant has 1 point for the presence of an increased STIR signal. Next, a total score of 48 is obtained from the six slices of the two SIJs. Then, 1 point is added for each SIJ exhibiting an intense signal (compared with the signal from adjacent blood vessels) or depth of BME ≥1 cm for the six slices, bringing the total score to 72.

### Clinical evaluation

The ASDAS [19] included back pain, general condition, and stiffness using a visual analog score, which was determined by doctor inquiry and patient self-reporting. C-reactive protein (CRP) and the erythrocyte sedimentation rate (ESR) were determined for all patients. For assessment of the ESR, 5 ml blood were obtained from the median antebrachial vein of each patient after a 12-hour fast and kept at 4 °C. Then, a 1.6 ml blood sample was combined with 0.4 ml sodium citrate (10^9^ mmol/l) and the mixture was placed into an ESR tube taking care not to create bubbles. The tube was placed vertically on the ESR stand and the ESR was read after an hour. For CRP, the blood sample underwent coagulation and was centrifuged. The serum was taken and kept at 4 °C. CRP levels were measured using a Roche Cobas 6000 c501 automatic analyzer (Roche Diagnostics, Laval, Canada) based on an immunoturbidimetry method, using the reagents and instructions provided by the manufacturer.

Treatment adverse effects were recorded based on the reports from the patients, clinical examination, and relevant biochemistry indexes.

### Statistical analysis

Factorial analysis was used for inter-group comparisons using SPSS 16.0 (IBM, Armonk, NY, USA). Intragroup comparisons were performed with a paired *t* test or rank sum test and nonparametric test. Two-sided *P* <0.05 was considered statistically significant.

## Results

Patient age ranged from 15 to 45 years; average patient disease duration was from 2 months to 10 years. Characteristics of the patients are presented in Table 1.

**Table 1:** Patient characteristics and disease parameters

	All	Anti-TNF alone	Anti-TNF + DMARDs	DMARDs alone
Age (years)	21 (15–36)	23 (15–29)	27 (18–32)	28 (22–36)
Gender (male/female)	12/44	3/15	6/15	3/14
Disease duration	2 (0.17–10)	0.67 (0.17–3)	2 (0.75–6)	7.5 (3.5–10)
HLA-B27-positive	91.1 % (51/56)	88.9 % (16/18)	90.5 % (19/21)	94.1 % (16/17)
Baseline				
SPARCC	32.45 ± 18.71	27.76 ± 18.38	39.53 ± 19.21	28.67 ± 15.51
ASDAS	2.84 ± 1.26	2.56 ± 1.24	3.58 ± 1.05	2.21 ± 1.03
ESR^a^	27.10 ± 32.39	27.94 ± 33.24	38.49 ± 38.50	12.14 ± 8.43
CRP^a^	17.50 ± 23.07	16.29 ± 23.05	26.29 ± 28.02	7.919 ± 5.60

^a^Not normally distributed, compared by paired rank test

*ASDAS* Ankylosing Spondylitis Disease Activity Indices, *CRP* C-reactive protein, *DMARD* disease-modifying anti-rheumatic drug, *ESR* erythrocyte sedimentation rate, *SPARCC* Spondyloarthritis Research Consortium Canada, *TNF* tumor necrosis factor

### MRI scans

**Fig. 1:**
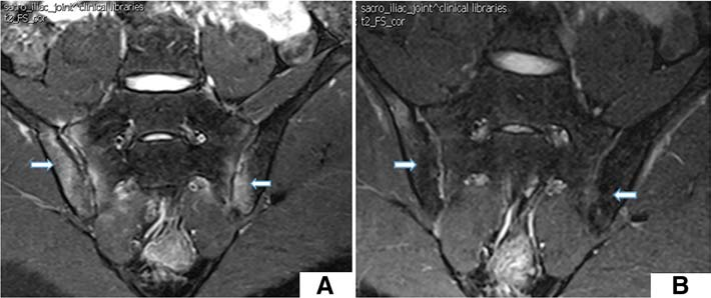
BME changes **a** before (SPARCC score 65) and **b** after 6 months of anti-TNFα monotherapy (SPARCC score 22)

**Fig. 2:**
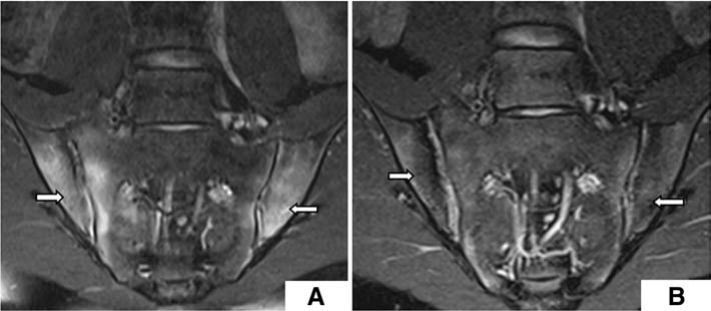
BME changes **a** before (SPARCC score 72) and **b** after 6 months of treatment with anti-TNFα combined with DMARDs (SPARCC score 13)

**Fig. 3:**
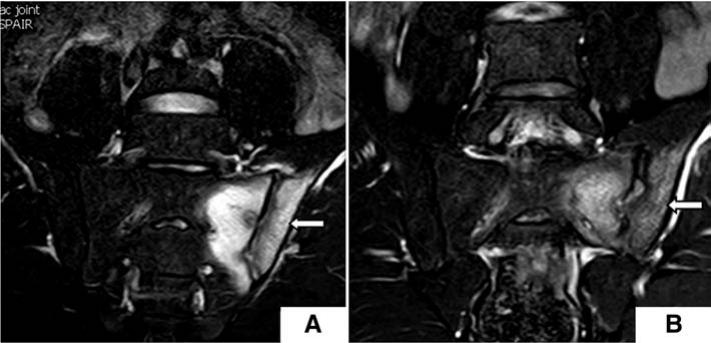
BME changes **a** before (SPARCC score 36) and **b** after 8 months of DMARDs monotherapy (SPARCC score 33)

For the seven (17.9 %) patients treated with anti-TNFα, BME completely disappeared within 12 months. Similar outcomes were observed in patients treated with anti-TNFα alone or in combination with DMARDs; for these patients, there was a significant decrease in their SPARCC scores within 12 months of treatment (*P* < 0.05) (Table 2 and Figs. 1 and 2). Nevertheless, for patients treated by DMARDs, SPARCC scores did not change after treatment (*P* = 0.419) (Table 2 and Fig. 3).

**Table 2:** Differences of indexes in patients with different treatment method

Index	Baseline (mean ± SD)	Post-treatment (mean ± SD)	*t/Z*	*P*
Patients treated by anti-TNF alone (*n* = 18)^a^				
SPARCC	27.76 ± 18.38	14.38 ± 14.91	2.681	**0.011**
ASDAS	2.56 ± 1.24	1.83 ± 0.71	4.231	**0.006**
ESR^b^	27.94 ± 33.24	5.72 ± 7.06	–2.763	**0.009**
CRP^b^	16.29 ± 23.05	2.70 ± 2.55	–2.470	**0.022**
Patients treated by anti-TNF combined with DMARDs (*n* = 21)^c^				
SPARCC	39.53 ± 19.21	20.71 ± 15.98	5.110	**0.000**
ASDAS	3.58 ± 1.05	1.88 ± 0.74	4.278	**0.003**
ESR^b^	38.49 ± 38.50	9.02 ± 13.29	–2.703	**0.010**
CRP^b^	26.29 ± 28.02	8.43 ± 15.79	–2.329	0.053
Patients treated by DMARDs alone (*n* = 17)^d^				
SPARCC	28.67 ± 15.51	21.66 ± 11.23	0.820	0.419
ASDAS	2.21 ± 1.03	1.99 ± 0.47	1.301	0.261
ESR^b^	12.14 ± 8.43	4.65 ± 4.27	–2.396	**0.013**
CRP^b^	7.919 ± 5.60	5.55 ± 4.46	–1.911	0.056

^a^Baseline of clinical practices from patients in anti-TNF group: 85.7 % male; age, 23.3 ± 6.43; symptom duration, 3.15 ± 2.98; HLA-B27-positive, 100 %

^b^Not normally distributed, compared by paired rank test

^c^Baseline of clinical practices from patients in combination group: 82.35 % male; age, 21.6 ± 3.36; symptom duration, 2.97 ± 2.69; HLA-B27-positive, 88.2 %

^d^Baseline of clinical practices from patients in DMARD group: 80 % male; age, 20.5 ± 2.75; symptom duration, 3.0 ± 2.66; HLA-B27-positive, 90 %

*ASDAS* Ankylosing Spondylitis Disease Activity Indices, *CRP* C-reactive protein, *DMARD* disease-modifying anti-rheumatic drug, *ESR* erythrocyte sedimentation rate, *SD* standard deviation, *SPARCC* Spondyloarthritis Research Consortium Canada, *TNF* tumor necrosis factor

Patients treated with anti-TNFα alone showed decreased SPARCC scores, ASDAS indices, ESR, and CRP within 12 months of starting treatment. SPARCC scores, ASDAS indices, and ESR were significantly decreased within 12 months after treatment with anti-TNFα combined with DMARD therapy. However, there were no significant differences in SPARCC scores, ASDAS indices, ESR, or CRP for patients treated with DMARDs alone within the 12-month study period (Table 3).

**Table 3:** Differences of indexes in patients at different treatment periods

Index	Baseline (mean ± SD)	Post-treatment (mean ± SD)	*t/Z*	*P*
Group A: 3 months of full-dose anti-TNFα (*n* = 10; *n* = 6 for anti-TNFα alone, *n* = 4 for combination)^a^				
ASDAS	3.43 ± 1.55	1.66 ± 0.40	2.675	**0.043**
SPARCC	31.67 ± 18.64	14.50 ± 18.41	3.768	**0.012**
ESR^b^	52.00 ± 43.83	5.00 ± 5.55	–2.023	**0.046**
CRP^b^	36.58 ± 40.37	1.92 ± 1.09	–1.782	0.083
Group B: 4–6 months of anti-TNFα (*n* = 17; *n* = 4 for anti-TNFα alone, *n* = 13 for combination)^c^				
ASDAS	2.46 ± 0.58	1.58 ± 0.69	4.920	**<0.001**
SPARCC	34.98 ± 18.93	18.54 ± 15.72	4.285	**0.001**
ESR^b^	27.77 ± 34.95	7.22 ± 12.31	–2.749	**0.007**
CRP^b^	16.34 ± 23.11	4.37 ± 17.87	–2.283	**0.025**
Group C: 9–12 months of anti-TNFα (*n* = 12; *n* = 6 for anti-TNFα alone, *n* = 6 for combination)^d^				
ASDAS	2.91 ± 0.89	2.05 ± 1.21	2.517	**0.042**
SPARCC	38.24 ± 15.39	23.09 ± 15.65	2.527	**0.038**
ESR^b^	28.29 ± 27.77	12.43 ± 13.57	–1.498	0.128
CRP^b^	14.43 ± 11.65	10.81 ± 16.54	–1.529	0.134

^a^Baseline of clinical practices from patients in Group A: 85.7 % male; age, 23 ± 5.62; symptom duration, 2.6 ± 3.25; HLA-B27-positive, 85.7 %

^b^Not normally distributed, compared by paired rank test

^c^Baseline of clinical practices from patients in Group B: 85.7 % male; age, 23.3 ± 6.43; symptom duration, 3.15 ± 2.98; HLA-B27-positive, 100 %

^d^Baseline of clinical practices from patients in Group C: 80 % male; age, 21.3 ± 2.53; symptom duration, 3.97 ± 3.52; HLA-B27-positive, 90 %

*ASDAS* Ankylosing Spondylitis Disease Activity Indices, *CRP* C-reactive protein, *DMARD* disease-modifying anti-rheumatic drug, *ESR* erythrocyte sedimentation rate, *SD* standard deviation, *SPARCC* Spondyloarthritis Research Consortium Canada, *TNFα* tumor necrosis factor alpha

**Fig. 4:**
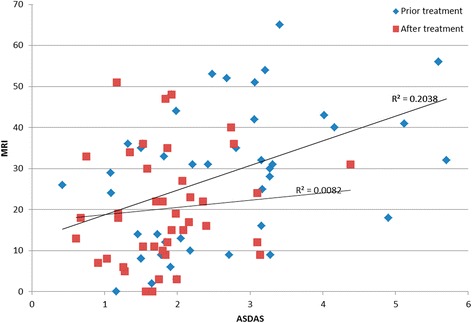
Correlations between ASDAS values and MRI values in all patients before treatment. *ASDAS* Ankylosing Spondylitis Disease Activity, *MRI* magnetic resonance imaging

In detail, SPARCC scores in Group A were significantly decreased after 3 months of full-dose anti-TNFα (*P* = 0.012). In Group B (4–6 months of full-dose anti-TNFα), SPARCC values were reduced (*P* = 0.001). In Group C, patients were treated 9–12 months of anti-TNFα, and the values were also reduced (*P* = 0.038). The correlation between ASDAS values and MRI values was assessed, showing good correlation pre treatment but not after treatment (Fig. 4).

### Adverse effects

Two patients experienced adverse reactions in the form of a temporary rash at the area of injection. One subject experienced a mild rash after oral sulfasalazine (SASP) intake. The diameters of the rashes were less than 5 mm, mild, without skin ulceration, and lasted for only 2 days without any treatment. One patient experienced a urinary tract infection during the second month of anti-TNFα treatment, caused by *Escherichia coli*. The patient was treated with levofloxacin for 2 weeks based on results from a bacterial culture. Two patients experienced mild liver function abnormalities (patient 1, ALT = 42 U/l; patient 2, AST = 57 U/l). Liver function returned to normal after 2 weeks of liver treatment.

## Discussion

NSAIDs and anti-rheumatic drugs are the primary treatment for SpA. SASP is believed to reduce the ESR and morning stiffness [20]. The pathogenesis of SpA is complex. Recent studies have revealed that TNFα plays a key role in the development of the disease. Studies have shown that TNF antagonists can significantly improve both disease activity and joint function [20–25].

In this study, patients with high SpA activity treated by targeted therapy (including etanercept, infliximab, and adalimumab) for 3 months showed significantly decreased ASDAS indices, SPARCC scores, and ESR (*P* < 0.05), indicating that 3 months of full-dose anti-TNFα therapy have significant efficacy as treatment against SpA. Furthermore, there was obvious resorption of SIJ acute inflammatory lesions observed from MRI examination. In 2013, an expert advisory committee advised the use of etanercept for the treatment of rheumatoid arthritis and AS [26], and suggested that assessment of treatment efficacy in patients with AS should be performed at least 3 months after starting the treatment. Based on the present study, anti-TNFα could be of great help in improving the symptoms of SpA. After 3 months of anti-TNFα, the clinical activity and BME of the SIJ in patients with SpA showed significant improvement, and patients might maintain remission with continued use, even with a reduction in treatment dose. As shown in this study, both anti-TNFα alone and in combination with DMARDs showed significant changes in SPARCC scores, ASDAS indices, and ESR. Previous studies [26–28] showed that there were differences of SPARCC score in disease-dormant patients and in disease-active patients. Furthermore, the SPARCC score has been proven to be positively correlated with the BASDAI, suggesting that the SPARCC scoring system can be used to assess inflammation activity in SpA. The SPARCC scoring system therefore plays an important role in evaluating treatment efficacy. The SPARCC scoring system provides a reference for imaging and evaluating the efficacy of different therapies. SIJ MRI examination has high sensitivity for the early stages of sacroiliitis [29], and it is safer and easier to repeat than X-ray or computed tomography scan.

There were some limitations to this study. The sample size was small and the timing of MRI examinations was not similar for all patients. Furthermore, the treatment dose levels varied between participants. In this study, DMARDs seem to have a modest effect at best on SpA, but this study was not designed to examine the efficacy of DMARDs. The results suggest that only a 3-month follow-up might be necessary to assess the response to treatment, and that subsequent follow-ups are relatively optional. However, this study was not designed to address this issue, and we cannot draw any conclusion on this point. Additional multicenter trials are necessary to determine adequately the usefulness of anti-TNFα and the SPARCC scoring system in patients with SpA.

## Conclusions

The SPARCC scoring system is a good index for showing the severity of sacroiliitis pre treatment. In addition, this study showed that anti-TNFα was effective in reducing BME of the SIJ examined by MRI.

## Abbreviations

ADC: Apparent diffusion coefficient
AS: Ankylosing spondylitis
ASAS/OMERACT: International Association for the Evaluation of Spinal Arthritis/Outcome Measures in Rheumatology
ASDAS: Ankylosing Spondylitis Disease Activity
BASDAI: Bath Ankylosing Spondylitis Disease Activity Index
BME: Bone marrow edema
CRP: C-reactive protein
DMARD: Disease-modifying anti-rheumatic drug
ESR: Erythrocyte sedimentation rate
EULAR: European League against Rheumatism
MRI: Magnetic resonance imaging
NSAID: Nonsteroidal anti-inflammatory drug
SIJ: Sacroiliac joint
SASP: Sulfasalazine
SpA: Spondyloarthritis
SPARCC: Spondyloarthritis Research Consortium Canada
STIR: Short-tau inversion recovery
T1WI: T1-weighted images
T2-fs: T2 fast-suppressed
TNFα: Tumor necrosis factor alpha
